# A *TOMM40/APOE* allele encoding *APOE*‐E3 predicts high likelihood of late‐onset Alzheimer’s disease in autopsy cases

**DOI:** 10.1002/mgg3.1317

**Published:** 2020-05-30

**Authors:** Selma M. Soyal, Markus Kwik, Ognian Kalev, Stefan Lenz, Greta Zara, Peter Strasser, Wolfgang Patsch, Serge Weis

**Affiliations:** ^1^ Institute of Pharmacology and Toxicology Paracelsus Medical University Salzburg Austria; ^2^ Division of Neuropathology Neuromed Campus, Kepler University Hospital Linz Austria; ^3^ Institute of Laboratory Medicine Paracelsus Medical University Salzburg Austria

**Keywords:** Alzheimer’ disease, APOE, beta‐amyloid, genetics, haplotypes, neurofibrillary tangles, TOMM40

## Abstract

**Background:**

The *APOE*‐ε4 allele is an established risk factor for Alzheimer's disease (AD). *TOMM40* located adjacent to *APOE* has also been implicated in AD but reports of *TOMM40* associations with AD that are independent of *APOE*‐ε4 are at variance.

**Methods:**

We investigated associations of AD with haplotypes defined by three *TOMM40* and two *APOE* single nucleotide polymorphisms in 73 and 71 autopsy cases with intermediate and high likelihood of AD (defined by BRAAK stages <V and V‐VI), respectively, and in 150 controls without major neurodegenerative diseases.

**Results:**

We observed eight haplotypes with a frequency >0.02. The two haplotypes encoding *APOE*‐E4 showed strong associations with AD that did not differ between intermediate and high likelihood AD. In contrast, a *TOMM40* haplotype encoding *APOE‐*E3 was identified as risk haplotype of high‐ (*p* = .0186), but not intermediate likelihood AD (*p* = .7530). Furthermore, the variant allele of rs2075650 located in intron 2 of *TOMM40*, increased the risk of high‐, but not intermediate likelihood AD on the *APOE*‐ε3/ε3 background (*p* = .0230).

**Conclusion:**

The striking association of *TOMM40* only with high likelihood AD may explain some contrasting results for *TOMM40* in clinical studies and may reflect an association with more advanced disease and/or suggest a role of *TOMM40* in the pathogenesis of neurofibrillary tangles.

## Graphical abstract

Associations of Alzheimer disease (AD) with haplotypes of TOMM40 and APOE were investigated. A TOMM40 haplotype encoding APOE‐E3 was identified as risk haplotype of high‐ (*p* = .0186), but not intermediate likelihood AD (*p* = .7530). The variant allele of rs2075650 located in intron 2 of TOMM40, increased the risk of high‐, but not intermediate likelihood AD on the APOE‐ε3/ε3 background (*p* = .0230). The striking association of TOMM40 only with high likelihood AD may explain some contrasting results for TOMM40 in clinical studies and may reflect an association with more advanced disease and/or suggest a role of TOMM40 in the pathogenesis of neurofibrillary tangles.

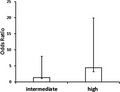

## 1 INTRODUCTION

Alzheimer's disease (AD) is the most common neurodegenerative disease worldwide. This progressive brain disease begins well before symptoms of cognitive impairment appear. The most common form of AD is late‐onset AD which develops after 60 years of age and accounts for more than 95% of AD cases. The neuropathological hallmarks of AD are progressive accumulations of plaques containing protein fragments (<43 amino acids) of beta‐amyloid (Aβ) outside of neurons and twisted strands of tau termed neurofibrillary tangles (NFTs) inside neurons. These changes are accompanied by damage and death of neurons (Querfurth & LaFerla, 2010; Scheltens et al., 2016; Selkoe, 2011). Up to 80% of dementia cases are caused by AD. In 2015, over 46 million people were estimated to live with dementia worldwide. This number is estimated to increase to 131.5 million by 2050. The risk of AD increases with the number of affected first‐degree relatives and genetic factors are thought to account for 58%–79% of the risk of developing AD (Gatz et al., 2006).

Among an increasing number of genes implicated in AD, the *APOE‐*ε4 allele confers the strongest and most often replicated risk of AD; while the *APOE*‐ε2 allele appears to be protective (Corder et al., 1993, 1994; Holtzman, Herz, & Bu, 2012; Strittmatter et al., 1993). At the *APOE* locus, three common alleles termed ε2, ε3, and ε4 exist that encode the three isoforms E2, E3, and E4 and specify six different geno‐ and phenotypes. The three isoforms differ by a single amino acid resulting from cysteine/arginine interchanges, encoded by two single nucleotide polymorphisms (SNPs) in exon 4 of *APOE*.

*APOE* is located in a linkage disequilibrium (LD) block on chromosome 19 that also includes *TOMM40, APOC1, APOC4, and APOC2*. SNPs in both *TOMM40* and *APOC1* have been linked with AD risk. Associations of rs4420638 located within *APOC1* and AD were entirely explained by its LD with *APOE* (Li et al., 2008). However, sequence variations within *TOMM40*, specifically rs2075650 and rs10524523, were suggested to afford AD risk stratification beyond the effects of the *APOE‐*ε4 allele, as they showed significant risk differences for AD or related phenotypes on the *APOE‐*ε3 allele background (Omoumi et al., 2014b; Roses et al., 2010). The latter results were confirmed in some (Case lli et al., 2012; Chung et al., 2013; Elias‐Sonnenschein et al., 2013; He et al., 2016; Horwitz, Lam, Chen, Xia, & Liu, 2019; Huang et al., 2016; Johnson et al., 2011; Li et al., 2013; Omoumi et al., 2014a; Potkin et al., 2009; Prendecki et al., 2018; Xiao et al., 2015; Yu et al., 2017) but not all studies (Cruchaga et al., 2011; Jun et al., 2012; Lyall et al., 2014).

It was suggested that allele A of *TOMM40* rs2075650 polymorphism was a risk factor for AD (odds ratio [OR] = 2.87, 95% confidence interval [CI]: 2.46–3.34, *p*‐value < .001). Alleles A of CD33 rs3865444 and A of *TOMM40* rs157580 were both protective factors for AD onset (OR = 0.94, 95% CI: 0.90–0.98, *p*‐value = .003; OR = 0.62, 95% CI: 0.57–0.66, *p*‐value < .001) (Bao, Wang, & Mao, 2016). The presence of an association between *TOMM40* SNPs and LOAD was reported in an Italian population (Bagnoli et al., 2013). Associations of three SNPs (rs157580, rs2075650, and rs11556505) with LOAD risk were observed in the investigated sample as well as in the non APOE ε4 carriers leading to the suggestion that *TOMM40* polymorphisms may play a role in the pathogenesis of LOAD in Han Chinese (Ma et al., 2013). In late onset AD (LOAD), SNPs near APOE gave highly significant results (e.g., rs2075650, *p* = 3.2 × 10^−81^), but no other genome‐wide significant evidence for association was found (Wijsman et al., 2011).

Significant SNPs associated with the Aβ_1‐42_ levels in cerebrospinal fluid (CSF) included rs2075650 in the intron region of *TOMM40* with a *p*‐value ≥ 1 × 10^–16^ (Souza, Araújo, Costa, & Oliveira, 2016). Significant differences in Minor allele frequency (*p* < .05, uncorrected) were seen for *CR1* (rs1408077; OR, 1.59; 95% CI, 1.01–2.49), *PICALM* (rs541458; OR, 0.68, 95% CI, 0.47–0.98), *TOMM40* (rs2075650; OR, 4.30; 95% CI, 2.61–7.06); and possession of 1 or more *APOE* ε4 alleles (OR, 9.84; 95% CI, 5.48–17.67) using CSF to replicate genetic associations in AD (Schott, 2012).

The effects of *TOMM40* rs2075650 on cognition have been described. Although variants of this SNP were not associated with poor cognitive performance (Cruz‐Sanabria et al., 2018), rs2075650 was associated with residualized delayed recall level at the genome‐wide level (*p* = 5.0 × 10^−8^) (Arpawong et al., 2017). SNP rs2075650 also correlated with the percentile of Rey Complex Figure Test copy score (*β* = 14.005, *p* corrected = 0.021) and the percentile of total score in phonemic fluency (*β* = 11.052, *p* corrected = 0.035) (Chung et al., 2014). In addition, SNP rs2075650 had a genome‐wide significant association with cognitive aging (*p* = 2.5 × 10^–8^) (Davies et al., 2014).

Neuro‐Imaging studies revealed that on structural magnetic resonance imaging, rs2075650 (TOMM40) was associated with changes of the right caudate (Moon et al., 2015) as well as with smaller HV hippocampal volume (HV) (*p* = .0054) (Chauhan et al., 2015), and with various imaging phenotypes in multiple regions of interests (Xu, Shen, & Pan, 2014). There was no effect of *APOE* and *TOMM40* on episodic memory performance and HV (Ferencz et al., 2013).

With regard to longevity three SNPs (rs2075650 [*TOMM40*], rs4420638 [*APOC1*], and rs429358 [*APOE*]) were significantly associated with survival to 90 years after correction for multiple testing (*p* < .001) (Shadyab et al., 2017). A haplotype analysis suggested that individuals carrying the haplotype A‐A‐A‐A‐T‐A‐T‐G‐C‐A (rs7254892‐rs157580‐rs2075649‐rs2075650‐rs157582‐rs8106922‐rs1160985‐rs405697‐rs439401‐rs445925) tended to have longer lifespan than those carrying the most common haplotype G‐G‐A‐A‐C‐A‐C‐A‐T‐G (OR = 1.59, 95% CI = 1.19–2.12, *p* = .0018, Pc = 0.0216). These findings indicated that variants in the *TOMM40/APOE/APOC1* region might be associated with human longevity (Lin et al., 2016). Rs2075650 in *TOMM40*, rs405509 in *APOE* and rs519825 in *PVRL2* showed a significant association with human longevity in a replication cohort (Lu et al., 2014). The TOMM40 locus (rs2075650) showed compelling evidence of association with human life span (*p* = 5.27 × 10^–4^) (Shi et al., 2012). The linked G allele in rs2075650 of *TOMM40* was associated with increased mortality (Schupf et al., 2013).

The role of *TOMM40* was investigated in another neurodegenerative disorder, that is, Fronto‐temporal lobe dementia. LD (*r* ^2^ = .35) between *TOMM40* (rs2075650) and *APOC1* (rs1064725) was observed in primary progressive aphasia (PPA), but not in controls and in behavioral variant fronto‐temporal dementia (bvFTD). Within this region of 26.9 kb, LD (*r* ^2^ ≥ .50) between *TOMM40* (rs2075650) and *APOE* (rs429358) linkage was observed in bvFTD and in controls, but not in PPA (Seripa et al., 2012).

The *TOMM40* rs2075650 G allele was a significant risk factor for lifetime depression (*p* = .00006) and, in depressed subjects, was a significant predictor of low extraversion (*p* = .009). The results suggest that *TOMM40* rs2075650 may be a risk factor for the development of depression characterized by reduced extraversion, impaired executive function, and decreased positive emotional recall, and reduced top‐down cortical control during sad emotion processing (McFarquhar et al., 2014).

In stroke, the rs2075650 (G → A) (*p* = .0102) of *TOMM40* (*p* = .0443; recessive model; OR = 0.50) and rs273909 of *SLC22A4* (*p* = .0123; dominant model; OR = 0.45) were significantly associated with ischemic stroke with the minor G and C allele, respectively, being protective against this condition (Yamase et al., 2015).

*TOMM40* and *APOE* variants are independently and additively associated with body mass index (BMI) whereby *TOMM40* (rs2075650) has an independent BMI‐lowering effect (Kulminski et al., 2019). SNP rs2075650 of *TOMM40* (*p* = .0004, OR, 1.43; dominant model) was significantly associated with hyper‑LDL‑cholesterolemia (Abe et al., 2015). Four additional AD risk SNPs were nominally associated with obesity (rs17125944 at *FERMT2*, pBMI = 4.03 × 10^–5^, pBMI corr = 2.50 × 10^–3^; rs3851179 at *PICALM*; pBMI = 0.002, rs2075650 at *TOMM40/APOE*, pBMI = 0.024, rs3865444 at *CD33*, pBMI = 0.024) (Hinney et al., 2014). The minor allele (G) (CAD risk allele) of rs2075650 (*TOMM40/APOE*) was associated with lower levels of high‐sensitivity C‐reactive protein (Christiansen, 2017).

We, therefore, addressed the issue of potential effects of *TOMM40* alleles that are independent of the *APOE‐*ε4 allele and compared the distributions of *TOMM40/APOE* haplotypes in neuropathologically characterized Caucasian postmortem samples of both AD cases and controls free of major neurodegenerative disorders.

## 2 METHODS

### 2.1 Autopsy samples and neuropathological examination

Human postmortem DNA samples from 144 AD cases and 150 controls without neuropathological findings indicative of neurodegenerative diseases were procured from the brain bank of the Division of Neuropathology, Neuromed Campus, Kepler University Hospital, Linz, Austria. Following federal law (BGBl. Nr. 1/1957, §25 KAKuG) and state regulation (LGBl.Nr. 40/1985), postmortem tissue can be removed upon autopsy for diagnostic or scientific purposes. Approval from the Ethics Committee of the State of Upper Austria for the use of postmortem tissue for molecular analysis is available (EK Nr: 1028/2017). Brains were removed within 24 hr after death of the patient. Upon removal, the fresh brain was separated into the two hemispheres by a mid‐sagittal cut which also allows the hemi‐dissection of the cerebellum and the brain stem. One hemisphere of the brain was immediately fixed in 4% formaldehyde for 1 week prior to neuropathological dissection and diagnosis. After thorough gross‐anatomical examination of the fixed hemisphere, 20 tissue blocks were cut for histopathological examination. For each block the following staining was performed: Hematoxylin and eosin, Luxol Fast Blue, immunohistochemistry for beta A4 amyloid, tau, phosphorylated tau, tau 3 repeat, tau 4 repeat, α‐synuclein, ubiquitin, p62, TDP‐43, and FUS. The other hemisphere was fresh‐frozen as follows: The brainstem with the cerebellum was dissected at the level of the pons and midbrain. A slice containing the substantia nigra and nucleus ruber was generated cranialward and frozen. The cerebellum was removed from the brainstem by a cut through the cerebellar peduncles. The brainstem was cut into thin slabs perpendicular to its main axis. Slices of the cerebellum were generated parallel to the vermis. The hemisphere was cut into 1 cm thick coronal slabs and frozen.

For the diagnosis of AD, the neuropathological criteria reported by the National Institute on Aging and the Reagan Institute Working Group (Hyman & Trojanowski, 1997) were used. The Thal stages of amyloid deposition as proposed in the revised 2012 version of the NIA criteria (Hyman et al., 2012) were not assessed as the collection of brains began in 2007. The CERAD score (A, B, C) was determined as well as the staging of NFTs following Braak & Braak (stage I‐II, III‐IV, and V‐VI) (Braak & Braak, 1991). Finally, the likelihood that dementia was caused by amyloid deposits and NFTs, that is, AD, was determined as published by the National Institute on Aging and the Reagan Institute Working Group (Hyman et al., 2012; Hyman & Trojanowski, 1997): (a) High likelihood with high density of neuritic plaques and NFTs, high CERAD score, Braak & Braak V/VI; (b) Intermediate likelihood with moderate density of neuritic plaques and NFTs, moderate CERAD score, Braak & Braak III/IV, and (c) Low likelihood with limited neuritic plaques and NFTs, low CERAD score, Braak & Braak I/II. In our sample of 144 AD cases, 71 and 73 brains fulfilled the criteria for high and intermediate likelihood, respectively.

### 2.2 Genotyping

Single nucleotide polymorphisms in APOE (rs429358 and rs7412) were selected to distinguish the ε2, ε3, and ε4 alleles. TOMM40 SNPs (rs157580, rs2075650, and rs8106922) were chosen because of their frequent use in previous studies with positive outcomes (www.alzgene.org) (Bertram, McQueen, Mullin, Blacker, & Tanzi, 2007).

For genotyping purposes, a small piece of tissue from the frontal pole was removed from which DNA was extracted. The QIAsymphony DSP Virus/Pathogen Mini Kit, Version 1 was used for DNA extraction on the fully automated QIAsymphony SP instrument (Qiagen) that performed the purification of nucleic acids or proteins using magnetic‐particle technology. DNA concentration was determined with the NanoDrop^TM^ spectrophotometer (Thermo Fisher Scientific). Taqman Genotyping Assays were used to type rs429358 and rs7412, respectively, in exon 4 of APOE. *APOE* genotyping was performed on an ABI7500 Fast Real‐Time PCR System (Applied Biosystems) using the allelic discrimination protocol according to the manufacturer's protocol. All runs included three nontemplate‐controls and a positive control sample for each genotype (ε2ε2, ε2ε3, ε3ε3, ε4ε4, ε4ε3, and ε3ε3 for rs429358 and rs7412) to assist with allele calling. Samples and controls were diluted to 10 ng/µl and a 2 µl sample was added to 18 µl Master Mix consisting of 10 µl 2x TaqMan Genotyping Master Mix, 0.5 µl 40x TaqMan Genotyping Assay and 7.5 µl PCR‐grade water (Applied Biosystems). Samples were amplified using the AB universal standard thermal cycling protocol (10 min activation at 95°C followed by 40 cycles of 15 s denaturation at 95°C and 1 min annealing‐extension at 60°C). After sample amplification, data collection for allelic discrimination was performed. Allele calling for ε4 and ε2 was done separately and the results of both calls were combined to determine the genotype. Samples that were inconclusive in one run were repeated. We also typed rs157580, 2075650, and rs8106922, all located in intronic sequences of *TOMM40*. The respective TaqMan Genotyping Assays (Applied Biosystems) were C_11466079_1, C_3084828_20, and C_11711485_10. The accuracy of typing was verified by sequencing of five alleles and repeat assays in 30 samples.

### 2.3 Statistical analysis

For comparison of categorical or continuous variables, we used a contingency χ^2^‐test or ANOVA, respectively. Allele frequencies were estimated by gene counting. Agreement with Hardy–Weinberg equilibrium was ascertained using a χ^2^ goodness‐of‐fit test. Differences in allele frequency and genotype distributions between AD cases and controls were calculated using a χ^2^ distribution. To assess associations of genotypes with AD, we estimated ORs with CIs by univariate logistic regression analysis. To provide separate ORs for each genotype, we used two dummy variables with the respective wild‐type as the reference as described (Esterbauer et al., 2001). Sex and age were included as covariates in multivariate logistic regression models. The THESIAS software was used to estimate standardized pair‐wise linkage disequilibria (LD) expressed in terms of D’ (http://gencanvas.ecgene.net/downloads). Haplotype frequencies and unadjusted and co‐variate adjusted haplotype‐phenotype parameters were estimated as ORs for each haplotype being present with a predicted frequency >2% by comparison to the most frequent haplotype. Adjustment for the number of haplotypes is included for the calculation of statistical significance by the software. To compare statistical models for haplotypes based on *APOE* SNPs with models based on the combined *TOMM40* and *APOE* SNPs, we used the log‐likelihood ratio test. Two‐sided *p*‐values < .05 were considered statistically significant.

## 3 RESULTS

Single nucleotide polymorphisms were determined in 66 male and 78 female AD postmortem cases and 92 male and 58 female postmortem controls. The proportion of cases was higher in females than in males (*p* = .008). The average age (*SD*) at death was 85 (6) and 73 (15) in cases and controls, respectively (*p* < .001). The genotypes associated with the five loci fulfilled Hardy–Weinberg expectations. Standardized pair‐wise LD, expressed as D′, and *R* ^2^ values ranged between −1.0 to 0.60 and 0.0146 to 0.3537, respectively (Table S1). Minor allele frequencies were 0.313 for rs157580, 0.150 for rs2075650, 0.433 for rs8106922, 0.141 for rs429358, and 0.097 for rs7412. Minor allele frequencies differed between cases and controls for rs2075650 (0.208 vs. 0.093, *p* < .0001) and rs429358 (0.208 vs. 0.077, *p* < .0001). Comparison of genotype frequencies between AD cases and controls revealed significant differences for rs2075650 and rs429358 (Table 1). Average ORs for heterozygosity of the rs2075650 variant allele relative to common allele homozygosity more than doubled and the average OR for homozygosity of the variant allele was increased ~10‐fold in both unadjusted and adjusted analyses. Average ORs for heterozygosity and variant allele homozygosity at rs429358 were approximately 3 and 11, respectively, in unadjusted analysis and increased even further in the adjusted analysis. ORs (95% CI) relative to the *APOE‐*ε3/3 genotype were 2.45 (1.33–4.53) for the ε3/4 and 10.08 (1.33–88.66) for ε4/4 genotypes in univariate analyses. Respective ORs were even higher in analyses adjusted for sex and age of death. ORs for the *APOE*‐ε2/2 and ‐ε2/3 tended to decrease, but the decrease was not significant. (Table S2). We next determined possible effects of the *TOMM40* SNPs on the *APOE*‐ε3/3 background. While rs157580 and rs8106922 showed no significant effects, heterozygosity at rs2075650 was associated with a threefold increase in risk of AD, but significance was not maintained after adjustment for sex and age (Table S3).

**TABLE 1:** *TOMM40* and *APOE* polymorphisms and associated risk of AD

Gene/SNP	Genotype	Controls		AD cases		*p* ^a^	Odds ratio (95% CI)		
		*N* = 150	Frequency	*N* = 144	Frequency		Univariate analysis^b^		Multivariate analysis^c^
*TOMM40*/rs157580	AA	63	0.4200	70	0.4861		1.00		1.00
	AG	74	0.4933	64	0.4444		0.77 (0.48–1.25)		0.98 (0.56–1.73)
	GG	13	0.0867	10	0.0694	.5060	0.69 (0.28–1.69)		0.53 (0.19–1.48)
*TOMM40*/rs2075650000	AA	123	0.8200	91	0.6319		1.00		1.00
	AG	26	0.1733	46	0.3194		2.39 (1.38–4.15)		2.76 (1.44–5.31)
	GG	1	0.0067	7	0.0486	.0006	9.46 (1.14–78.25)		13.54 (1.36–134.87)
*TOMM40*/rs81069222	AA	44	0.2933	55	0.3819		1.00		1.00
	AG	69	0.4600	66	0.4583		0.76 (0.45–1.29)		0.72 (0.39–1.34)
	GG	37	0.2467	23	0.1597	.1089	0.50 (0.26–0.96)		0.31 (0.14–0.66)
*APOE*/rs429358	TT	128	0.8533	92	0.6389		1.00		1.00
	TC	21	0.1400	44	0.3056		2.91 (1.62–5.23)		5.84 (2.71–12.61)
	CC	1	0.0067	8	0.0556	.00006	11.13 (1.37–90.53)	29.14 (3.07–281.56)	
*APOE*/rs7412	CC	121	0.8067	119	0.8264		1.00	1.00	
	CT	27	0.1800	24	0.1667		0.90 (0.49–1.65)	0.69 (0.35–1.36)	
	TT	2	0.0133	1	0.0069	.8170	0.51 (0.05–5.68)	0.60 (0.03–11.21)	

Abbreviation: AD, Alzheimer's disease; CI, confidence interval; SNP, single nucleotide polymorphisms.

^a^ χ^2^ analysis.

^b^ Logistic regression.

^c^ Logistic regression adjusted for sex and age.

We next used haplotype analyses, as their information content is superior to that of SNPs. This has been demonstrated in numerous studies (Barendse, 2011; Khankhanian, Gourraud, Lizee, & Goodin, 2015; Shim, Chun, Engelman, & Payseur, 2009).

Out of the 32 possible haplotypes, eight haplotypes with an estimated frequency >0.02 were observed (Table 2). Univariate and sex‐adjusted analyses revealed a highly significant global haplotype effect in both the unadjusted and adjusted analyses (*χ* ^2^ = 29.8 and 35.2, 7 DF, *p* = .0001). As expected, ORs of haplotypes 11121 and 12121, both coding for E4, were increased in both unadjusted and adjusted analyses. The OR for haplotype 12111 encoding E3 was also significantly increased to ~3 in the unadjusted analysis, but the significance of the 12111 haplotype was not maintained after adjustment for sex and age. For each of the eight haplotypes, the squared correlation between true and predicted haplotype dose was >0.8. We next compared a model containing only the three occurring *APOE* haplotypes as predictors with the full model containing the eight *TOMM40/APOE* haplotypes. These analyses showed no significant superiority of the full model (*p* = .0905).

**TABLE 2:** *TOMM40/APOE* haplotypes and risk of AD

Haplotype	Frequencies (%)		Odds ratio (95% CI)			
	Control (*N* = 150)	AD cases (*N* = 144)	Univariate analysis	*p*	Multivariate analysis^a^	*p*
11211	0.4523	0.3558	1.00	reference	1.00	reference
11111	0.0363	0.0259	0.96 (0.31–2.93)	.9359	1.33 (0.37–4.78)	.6588
11112	0.0584	0.0373	0.82 (0.34–1.95)	.6477	0.87 (0.34–2.22)	.7755
11121	0.0204	0.0773	4.35 (1.62–11.68)	.0035	8.65 (2.09–35.73)	.0029
12111	0.0227	0.0628	3.20 (1.18–8.72)	.0227	2.80 (0.92–8.59)	.0710
12121	0.0562	0.1310	2.72 (1.38–5.37)	.0040	5.10 (2.24–11.63)	.0001
21111	0.2901	0.2275	0.91 (0.59–1.39)	.6536	1.21 (0.72–2.03)	.4558
21112	0.0354	0.0440	1.57 (0.58–4.26)	.3737	1.43 (0.45–4.51)	.5394

Note

For haplotype designation, 1 or 2 refers to the major or minor alleles, respectively, in the following order: rs157580, rs2075650, rs8106922 (all within *TOMM40*) and rs429358, rs7412 (within *APOE*); global haplotype effects for univariate and multivariate analyses *p* < .0001.

Abbreviation: AD, Alzheimer's disease; CI, confidence interval.

^a^ Adjusted for sex and age.

**FIGURE 1:**
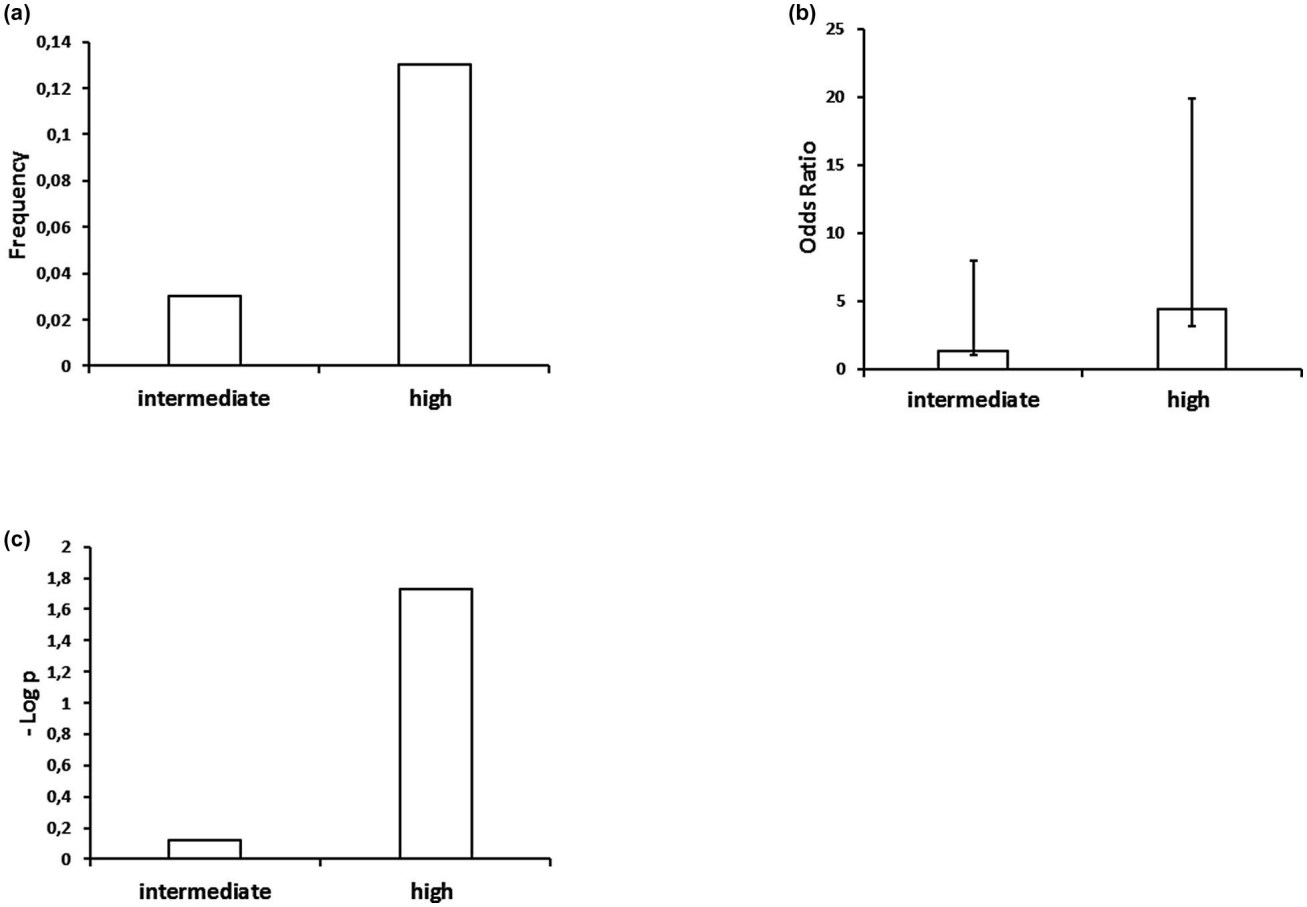
Plots showing the frequencies (a), Odds ratios (b), and *p*‐values (c) for the critical haplotype 12111 in the comparison between controls and intermediate or high likelihood of AD. AD, Alzheimer's disease

Approximately one half of the AD cases fulfilled the criteria for intermediate‐ and the other half for high likelihood AD (Table S4). To gain further insight into the association of *TOMM40* and AD, we compared geno‐ and haplotypes between controls and each of the two AD strata (Tables 3 and 4). In the comparison with intermediate likelihood cases, only the distribution of genotypes associated with rs429358 was different between cases and controls (*p* = .0015, Table S5). However, in the comparison with high likelihood cases, differences in the distribution of genotypes associated with rs2075650 (*p* < .00003) and rs429358 (*p* = .0002) were observed (Table S6). On the *APOE*‐ε3/3 background, genotypes associated with rs2075650 differed in the comparison with high‐ (*p* = .0022), but not with intermediate likelihood AD (*p* = .6155) (Tables S7 and S8) and ORs for carriers of the 2075650 G allele were 5.25 (1.98–14.29) in crude and 3.55 (1.19–10.56) in the age and sex adjusted analyses. Unadjusted and adjusted analyses of haplotypes revealed that the frequencies of the two E4 encoding haplotypes (11121 and 12121) were increased in cases with intermediate likelihood AD (OR 4.93, *p* = .0043 and OR 11.08, *p* = .0018, respectively) as well as in cases with high likelihood AD (OR 4.23, *p* = .0125 and OR 4.71, *p* = .0043) in comparison to controls. The frequencies of the ε2 encoding haplotypes 11112 and 21112 were not different. Furthermore, frequencies of haplotypes 11121 and 12121 did not differ between cases with intermediate and high AD likelihood (*p* = .9691 and 0.4130, respectively). However, the frequency of the E3 encoding haplotype 12111 was similar between controls and cases with intermediate likelihood of AD (OR 1.39, *p* = .6602), but higher than in controls in the comparison with high likelihood AD cases (OR 5.62, *p* = .0021) (Figure 1a–c) and significance was maintained after adjustment for age and sex (OR 4.45, *p* = .0186) and also by further adjustment for the comparison between intermediate and high likelihood by the Bonferroni correction (*p* = .0372). In addition, the model containing the eight *TOMM40/APOE* haplotypes was superior in predicting global haplotype effects than the model containing only the three haplotypes defined by the *APOE* SNPs (*χ* ^2^ = 18.442, 5 DF, *p < *.0032).

**TABLE 3:** *TOMM40*/*APOE* haplotypes and intermediate likelihood of AD

Haplotype	Frequencies (%)		Odds ratio (95% CI)			
	Control (*N* = 150)	Intermediate AD risk cases (*N* = 73)	Univariate analysis	*p*	Multivariate analysis^a^	*p*
11211	0.4521	0.3606	1.00		1.00	
11111	0.0364	0.0041	0.48 (0.01–17.36)	.6862	0.65 (0.02–20.76)	.8122
11112	0.0582	0.0474	1.06 (0.37–3.02)	.9073	1.17 (0.39–3.47)	.7778
11121	0.0204	0.0731	4.93 (1.65–14.77)	.0043	11.08 (2.44–50.19)	.0018
12111	0.0228	0.0241	1.39 (0.32–5.96)	.6602	1.30 (0.25–6.68)	.7530
12121	0.0563	0.1186	2.66 (1.22–5.7)	.0137	5.13 (2.08–12.66)	.0004
21111	0.2905	0.2743	1.18 (0.69–2.01)	.5356	1.50 (0.81–2.77)	.2018
21112	0.0352	0.0535	2.02 (0.67–6.12)	.2107	1.71 (0.47–6.18)	.4118

Note

For haplotype designation, 1 or 2 refers to the major or minor alleles, respectively, in the following order: rs157580, rs2075650, rs8106922 (all within *TOMM40*), and rs429358, rs7412 (within *APOE*); global haplotype effects *p* < .013 and *p* < .0002 for univariate and multivariate analyses.

Abbreviation: AD, Alzheimer's disease; CI, confidence interval.

^a^ Adjusted for sex and age.

**TABLE 4:** *TOMM40/APOE* haplotypes and high likelihood of AD

Haplotype	Frequencies (%)		Odds ratio (95% CI)			
	Controls (*N* = 150)	High AD risk cases (*N* = 71)	Univariate analysis	*p*	Multivariate analysis^a^	*p*
11211	0.4531	0.3506	1.00		1.00	
11111	0.0360	0.0523	2.40 (0.73–7.91)	.1499	3.01 (0.70–12.98)	.1370
11112	0.0585	0.0248	0.54 (0.16–1.88)	.3343	0.59 (0.16–2.20)	.4302
11121	0.0204	0.0799	4.23 (1.36–13.11)	.0125	8.14 (1.25–52.92)	.0281
12111	0.0227	0.1013	5.62 (1.87–16.86)	.0021	4.45 (1.28–15.43)	.0186
12121	0.0563	0.1454	2.98 (1.29–7.02)	.0122	4.71 (1.62–13.63)	.0043
21111	0.2898	0.1770	0.66 (0.36–1.19)	.1633	1.01 (0.51–1.97)	.9851
21112	0.0360	0.0363	1.30 (0.31–5.50)	.7177	1.08 (0.22–5.28)	.9190

Note

For haplotype designation, 1 or 2 refers to the major or minor alleles, respectively, in the following order: rs157580, rs2075650, rs8106922 (all within *TOMM40*) and rs429358, rs7412 (within *APOE*); global haplotype effects *p* < .0001 for univariate and multivariate analyses.

Abbreviation: AD, Alzheimer's disease; CI, confidence interval.

^a^ Adjusted for sex and age.

As *APOE* has been identified as a longevity locus (Deelen et al., 2011), we also determined associations of the 5 SNPs with age of death in AD cases. Genotypes associated with rs429358 and harboring one or two variant alleles decreased the age of death (Table 5). Haplotype analysis revealed a significant global effect (*p* = .0007). Haplotype 12121 (coding for E4) was associated with a 3.9 year earlier age of death per haplotype dose in comparison to the reference haplotype 11211 (*χ* ^2^, 7 DF, *p* < .0001), while haplotype 11121, the other E4 encoding haplotype, was associated with a 1.7 year reduction of age of death (*p* = .3312).

**TABLE 5:** Associations of SNPs in *TOMM40* and *APOE* with age of death in AD

Gene/SNP	Age of Death^a^			*p*
	Homozygous	Heterozygous	Variant	
*TOMM40*/rs157580	84.9 (6.8)	85.7 (5.7)	89.9 (4.6)	.0546
*TOMM40*/rs2075650	86.2 (5.8)	84.8 (6.8)	81.2 (8.0)	.0834
*TOMM40*/rs8106922	84.8 (7.2)	85.8 (5.1)	86.9 (6.9)	.3876
*APOE*/rs429358	86.7 (6.0)	84.3 (5.9)	79.0 (6.6)	.0009
*APOE*/rs7412	86.0 (6.3)	85.0 (6.3)	88.6 (0)	.7144

Abbreviation: AD, Alzheimer's disease; SNP, single nucleotide polymorphisms.

^a^ Results are means (*SD*) of years; ANOVA, adjusted for sex.

## 4 DISCUSSION

Our study strongly suggests that the *TOMM40* locus contributes to the risk of AD. Carriers of the rs2075650 variant allele increased the risk of high likelihood AD in cases with *APOE*‐ε3/3 genotype. In addition, a risk haplotype for high likelihood AD defined by three SNPs within *TOMM40* and coding for the *APOE‐*E3 isoform was identified. In the comparison of controls with all AD cases, similar associations were found in the crude analyses, but were not maintained after adjustment for sex and age of death. These results confirm and extend other reports showing an *APOE‐*ε4 allele independent effect of *TOMM40* in AD or related phenotypes (Li et al., 2013; Omoumi et al., 2014b; Roses et al., 2010; Yu et al., 2017). However, the transcriptional regulation of both *TOMM40* and *APOE* is complex and includes cis‐regulatory elements as well as epigenetic modifications in adjacent genes (Bekris, Lutz, & Yu, 2012; Shao et al., 2018). Hence, our data do not entirely exclude an effect of *APOE* expression. Interestingly, an integrated analysis of GWAS data along with expression and methylation quantitative trait loci (QTL) data revealed associations of AD with *TOMM40* that likely was mediated by effects on its expression and methylation (Marioni et al., 2018). In addition, an *APOE* independent role of *TOMM40* is supported by pathway analyses of GWAS data in a French AD case‐control study (Hong, Alexeyenko, Lambert, Amouyel, & Prince, 2010). Enrichment of genes annotated as being involved in intracellular protein transport was observed in AD. A total of 18 genes including genes encoding nucleoporins and several mitochondrial proteins contributed to the gene signature of AD. Among the genes in LD with *APOE*, only *TOMM40*, but not *APOE* showed significant direct and indirect connectivity to several genes in the protein transport pathway. Moreover, a recent study identified an association of the *PPARGC1A* encoding PGC‐1α with AD (Baker et al., 2019). PGC‐1α is a transcriptional co‐activator that controls mitochondrial biogenesis and function and activates genes involved in ROS defense. We have preliminary evidence that it also coactivates the transcription of *TOMM40* in neuroblastoma cells. (Kwik M., Patsch W., Soyal S.M., unpublished observation).

A striking finding of our study was the strong association of the *TOMM40/APOE* haplotype 12111 encoding the *APOE*‐E3 isoform with high likelihood AD (as defined by Braak stages V/VI), while a significant association with intermediate likelihood AD was not observed. This is in stark contrast with the *TOMM40/APOE*‐E4 encoding haplotypes that were associated with both intermediate and high likelihood AD and may reflect the deficient clearance of Aβ by the E4 protein (Holtzman et al., 2012). Importantly, an association of the *TOMM40* intron 6 poly‐T polymorphism rs10534523 with increased neuritic tangles and a higher likelihood of pathologically diagnosed AD in 168 autopsy cases with the *APOE‐*ε3/3 genotype has been reported previously (Li et al., 2013). Our data are in line with this report and extend its findings in that the *TOMM40* haplotype 12111 was predictive for high likelihood AD in a sample containing all common *APOE* genotypes. These results may help to explain the contrasting results on the link of *TOMM40* with clinically diagnosed AD, in whom Braak & Braak stages were not ascertained. Our study outcome may simply reflect more advanced disease. However, like the study by Li et al. (2013), our data may implicate *TOMM40* in the pathogenesis of NFT and may, therefore, be relevant for the pathogenesis of AD. According to the amyloid cascade hypothesis, the aggregation of Aβ peptides is thought to trigger the formation of insoluble tau aggregates (Hardy & Selkoe, 2002). Indeed, several pathways whereby Aβ facilitates tau pathology have been proposed (Stancu, Vasconcelos, Terwel, & Dewachter, 2014), but in the absence of a clear mechanism, the relevance of studies in cell and animal models has not been established in humans. We propose that *TOMM40* may contribute to the connection between Aβ and tau pathology. *TOMM40* encodes a beta‐barrel protein that forms the central pore in the outer mitochondrial membrane and is essential for the translocation of nuclear encoded proteins (Shiota et al., 2015). Aβ peptides and their precursor protein accumulate in mitochondria of AD patients and AD mouse models (Caspersen et al., 2005; Manczak et al., 2006). Several mechanisms whereby Aβ which is devoid of a canonical mitochondrial targeting sequence is translocated to mitochondria have been suggested (Cenini, Rub, Bruderek, & Voos, 2016; Hansson Petersen, 2008). Recently, recognition of Aβ_1‐42_ by TOMM22 and translocation via TOMM40 has been reported (Hu, Wang, & Zheng, 2018). As the recognition by TOMM22 involves a C‐terminal helical region of Aβ_1‐42_, not present in Aβ_1‐40_, the uptake may be selective for the more amyloidogenic peptide. Aβ_1‐42_ interacts with many mitochondrial proteins resulting in mitochondrial dysfunction and the generation of reactive oxygen species (Hernandez‐Zimbron et al., 2012). Transport of mitochondria along microtubules and removal of dysfunctional mitochondria are essential for proper energy metabolism of neurons (Sheng, 2014). Indeed, mitochondrial dysfunction and defective mitochondrial transport is an established pathology in AD (Calkins, Manczak, Mao, Shirendeb, & Reddy, 2011; Du et al., 2010). As tau, but not phosphorylated tau inhibits axonal transport of mitochondria, its hyperphosphorylation may reflect a futile and deleterious rescue mechanism to maintain elimination of defective mitochondria. Indeed, defective mitochondrial quality control induced by deletion of the protease subunit AFG3L2 causes hyperphosphorylation of tau in murine cortical neurons (Kondadi et al., 2014) and loss of axonal mitochondria resulting from ablation of Milton increases tau phosphorylation at an AD‐relevant site (Iijima‐Ando et al., 2012).

Bioinformatic analyses showed that rs2075650 is conserved and is part of three signatures of promoter histone marks and enhancer histone markers in six cell types (Kraja et al., 2014), has been identified as regulatory SNP, and is positioned in the binding site for Ras‐Responsive Element‐Binding Protein 1 (RREB1). RREB1 is a zinc finger transcription factor that binds to the RNA polymerase II core promoter and enhances the expression of NeuroD (Ray, Nishitani, Petry, Fessing, & Leiter, 2003). Moreover, predicted binding motifs for eight additional transcription factors are altered by this SNP (Farre et al., 2003). The other *TOMM40* SNPs included rs157580, also located in intron 2, and rs8106922, located in intron 6. The sequence harboring rs8106922 is only preserved in rhesus monkeys, but not in other higher vertebrates. In silico analysis identified no transcription factor binding site in this region. rs157580 is positioned in a sequence that is not at all conserved. It is adjacent to putative conserved binding sites for PPARγ and ERRγ, and the variant allele causes loss of one of two predicted binding sites for NRF2. NRF2 is a transcriptional target of PGC‐1α and plays a major role in mitochondrial biology. Deficiency of NRF2 has been shown to recapitulate transcriptomic changes in AD patients and to worsen APP and TAU pathology (Rojo et al., 2017). Furthermore, rs157580 is contained in a putative SMAD binding site (Khan et al., 2018).

Consistent associations of *APOE* with longevity have been observed (Christensen, Johnson, & Vaupel, 2006). rs2705650 was associated with longevity in a GWAS study (Deelen et al., 2011). Additional typing of the two SNPs defining the allele status of *APOE* was included and showed that *APOE* accounted for the effect of rs2705650 on aging. *APOE‐*ε4 carriers have an increased risk of cardiovascular disease (Eichner et al., 1993) that may contribute to earlier death. In our study, the *APOE* allele status was also a predictor of age of death in AD. However, our haplotype analysis suggested that only one of the two E4 encoding haplotypes was associated with lower age of death. Interestingly, this haplotype was also defined by the *TOMM40* risk allele 121. If confirmed in additional larger studies, this result would suggest that *TOMM40* may have a modulating effect on age of death.

The strengths of the paper include use of genetic analysis of haplotypes, an understudied area for AD and use of autopsy confirmed phenotypes. Limitations of our study include the modest sample size and the lower age at death in controls. Even though comparable results have been reported (Li et al., 2013), the lack of a replication data set limits the interpretations of the study results. In addition, our data provide no information as to which *TOMM40* SNPs (or SNPs in strong LD with the typed SNPs) is/are the causative polymorphism(s).

## 5 CONCLUSION

This study replicates and refines results on a role of *TOMM40* in AD and found an association of a *TOMM40/APOE* haplotype encoding the E3 isoform with high‐, but not with intermediate likelihood AD in autopsy cases. In contrast, the two *TOMM40/APOE* haplotypes encoding the E4 isoprotein revealed comparably strong associations with both high‐ and intermediate likelihood AD. Furthermore, the ε4 allele was a predictor of earlier death in AD cases. These results may help to explain the disagreement of earlier clinical studies on *TOMM40* as an independent risk factor and may be relevant for the pathogenesis of AD. However, additional studies are needed to confirm our results and provide mechanistic insight.

## CONFLICT OF INTEREST

The authors declare no competing interests.

## AUTHOR CONTRIBUTION

The neuropathologic diagnosis was rendered by OK and SW; DNA extraction and ApoE genotyping was performed by SL, Genotyping for TOMM40 was done by SMS, MK, GZ, and PS. Statistical analyses were done by PW. The paper was written by SMS, WP, and SW. Proof reading before submission was done by all authors.

## Supporting information

Table S1‐S8

## Data Availability

The data that support the findings of this study are available from the corresponding author upon reasonable request.

## References

[mgg31317-bib-0001] Abe, S. , Tokoro, F. , Matsuoka, R. , Arai, M. , Noda, T. , Watanabe, S. , … Yamada, Y. (2015). Association of genetic variants with dyslipidemia. Molecular Medicine Reports, 12, 5429–5436. 26238946 10.3892/mmr.2015.4081

[mgg31317-bib-0002] Arpawong, T. E. , Pendleton, N. , Mekli, K. , McArdle, J. J. , Gatz, M. , Armoskus, C. , … Prescott, C. A. (2017). Genetic variants specific to aging‐related verbal memory: Insights from GWASs in a population‐based cohort. Molecular Psychiatry, 12, e0182448 10.1371/journal.pone.0182448 PMC5553750 28800603

[mgg31317-bib-0003] Bagnoli, S. , Piaceri, I. , Tedde, A. , Bessi, V. , Bracco, L. , Sorbi, S. , & Nacmias, B. (2013). TOMM40 polymorphisms in Italian Alzheimer's disease and frontotemporal dementia patients. Neurological Sciences, 34, 995–998. 10.1007/s10072-013-1425-6 23546992

[mgg31317-bib-0004] Baker, E. , Sims, R. , Leonenko, G. , Frizzati, A. , Harwood, J. C. , & Grozeva, D. (2019). Gene‐based analysis in HRC imputed genome wide association data identifies three novel genes for Alzheimer's disease. PLoS ONE, 14, e0218111. 31283791 10.1371/journal.pone.0218111 PMC6613773

[mgg31317-bib-0005] Bao, J. , Wang, X. J. , & Mao, Z. F. (2016). Associations between genetic variants in 19p13 and 19q13 regions and susceptibility to Alzheimer disease: A meta‐analysis. Medical Science Monitor, 22, 234–243. 10.12659/msm.895622 26795201 PMC4727495

[mgg31317-bib-0006] Barendse, W. (2011). Haplotype analysis improved evidence for candidate genes for intramuscular fat percentage from a genome wide association study of cattle. PLoS ONE, 6, e29601 10.1371/journal.pone.0029601 22216329 PMC3247274

[mgg31317-bib-0007] Bekris, L. M. , Lutz, F. , & Yu, C. E. (2012). Functional analysis of APOE locus genetic variation implicates regional enhancers in the regulation of both TOMM40 and APOE. Journal of Human Genetics, 57, 18–25. 10.1038/jhg.2011.123 22089642 PMC3266441

[mgg31317-bib-0008] Bertram, L. , McQueen, M. B. , Mullin, K. , Blacker, D. , & Tanzi, R. E. (2007). Systematic meta‐analyses of Alzheimer disease genetic association studies: The AlzGene database. Nature Genetics, 39, 17–23. 10.1038/ng1934 17192785

[mgg31317-bib-0009] Braak, H. , & Braak, E. (1991). Neuropathological stageing of Alzheimer‐related changes. Acta Neuropathologica, 82, 239–259. 10.1007/bf00308809 1759558

[mgg31317-bib-0010] Calkins, M. J. , Manczak, M. , Mao, P. , Shirendeb, U. , & Reddy, P. H. (2011). Impaired mitochondrial biogenesis, defective axonal transport of mitochondria, abnormal mitochondrial dynamics and synaptic degeneration in a mouse model of Alzheimer's disease. Human Molecular Genetics, 20, 4515–4529. 10.1093/hmg/ddr381 21873260 PMC3209824

[mgg31317-bib-0011] Caselli, R. J. , Dueck, A. C. , Huentelman, M. J. , Lutz, M. W. , Saunders, A. M. , Reiman, E. M. , & Roses, A. D. (2012). Longitudinal modeling of cognitive aging and the TOMM40 effect. Alzheimer's & Dementia, 8, 490–495. 10.1016/j.jalz.2011.11.006 PMC3483561 23102119

[mgg31317-bib-0012] Caspersen, C. , Wang, N. , Yao, J. , Sosunov, A. , Chen, X. , Lustbader, J. W. , … Yan, S. D. (2005). Mitochondrial Abeta: A potential focal point for neuronal metabolic dysfunction in Alzheimer's disease. The FASEB Journal, 19, 2040–2041. 10.1096/fj.05-3735fje 16210396

[mgg31317-bib-0013] Cenini, G. , Rub, C. , Bruderek, M. , & Voos, W. (2016). Amyloid beta‐peptides interfere with mitochondrial preprotein import competence by a coaggregation process. Molecular Biology of the Cell, 27, 3257–3272. 10.1091/mbc.e16-05-0313 27630262 PMC5170859

[mgg31317-bib-0014] Chauhan, G. , Adams, H. H. H. , Bis, J. C. , Weinstein, G. , Yu, L. , Töglhofer, A. M. , … Debette, S. (2015). Association of Alzheimer's disease GWAS loci with MRI markers of brain aging. Neurobiology of Aging, 36, 1765.e7–1765.e16. 10.1016/j.neurobiolaging.2014.12.028 PMC4391343 25670335

[mgg31317-bib-0015] Christensen, K. , Johnson, T. E. , & Vaupel, J. W. (2006). The quest for genetic determinants of human longevity: Challenges and insights. Nature Reviews Genetics, 7, 436–448. 10.1038/nrg1871 PMC2726954 16708071

[mgg31317-bib-0016] Christiansen, M. K. (2017). Coronary artery disease‐associated genetic variants and biomarkers of inflammation. PLoS ONE, 12, e0180365. 28686695 10.1371/journal.pone.0180365 PMC5501546

[mgg31317-bib-0017] Chung, S. J. , Kim, M. J. , Kim, Y. J. , Kim, J. , You, S. , Jang, E. H. , … Lee, J. H. (2014). CR1, ABCA7, and APOE genes affect the features of cognitive impairment in Alzheimer's disease. Journal of the Neurological Sciences, 339, 91–96. 10.1016/j.jns.2014.01.029 24530172

[mgg31317-bib-0018] Chung, S. J. , Lee, J. H. , Kim, S. Y. , You, S. , Kim, M. J. , Lee, J. Y. , & Koh, J. (2013). Association of GWAS top hits with late‐onset Alzheimer disease in Korean population. Alzheimer Disease and Associated Disorders, 27, 250–257. 10.1097/wad.0b013e31826d7281 22975751

[mgg31317-bib-0019] Corder, E. H. , Saunders, A. M. , Risch, N. J. , Strittmatter, W. J. , Schmechel, D. E. , Gaskell, P. C. , … Pericak‐Vance, M. A. (1994). Protective effect of apolipoprotein E type 2 allele for late onset Alzheimer disease. Nature Genetics, 7, 180–184. 10.1038/ng0694-180 7920638

[mgg31317-bib-0020] Corder, E. H. , Saunders, A. M. , Strittmatter, W. J. , Schmechel, D. E. , Gaskell, P. C. , Small, G. W. , … Pericak‐Vance, M. A. (1993). Gene dose of apolipoprotein E type 4 allele and the risk of Alzheimer's disease in late onset families. Science, 261, 921–923. 10.1126/science.8346443 8346443

[mgg31317-bib-0021] Cruchaga, C. , Nowotny, P. , Kauwe, J. S. , Ridge, P. G. , Mayo, K. , & Bertelsen, S. ; … (2011). Association and expression analyses with single‐nucleotide polymorphisms in TOMM40 in Alzheimer disease. Archives of Neurology, 68, 1013–1019. 21825236 10.1001/archneurol.2011.155 PMC3204798

[mgg31317-bib-0022] Cruz‐Sanabria, F. , Bonilla‐Vargas, K. , Estrada, K. , Mancera, O. , Vega, E. , Guerrero, E. , … Pardo, R. (2018). Analysis of cognitive performance and polymorphisms of SORL1, PVRL2, CR1, TOMM40, APOE, PICALM, GWAS_14q, CLU, and BIN1 in patients with mild cognitive impairment and cognitively healthy controls. Neurologia, S0213‐4853(18)30198‐1. 10.1016/j.nrl.2018.07.002 34752346

[mgg31317-bib-0023] Davies, G. , Harris, S. E. , Reynolds, C. A. , Payton, A. , Knight, H. M. , Liewald, D. C. , … Deary, I. J. (2014). A genome‐wide association study implicates the APOE locus in nonpathological cognitive ageing. Molecular Psychiatry, 19, 76–87. 10.1038/mp.2012.159 23207651 PMC7321835

[mgg31317-bib-0024] Deelen, J. , Beekman, M. , Uh, H. W. , Helmer, Q. , Kuningas, M. , Christiansen, L. , … Slagboom, P. E. (2011). Genome‐wide association study identifies a single major locus contributing to survival into old age; the APOE locus revisited. Aging Cell, 10, 686–698. 10.1111/j.1474-9726.2011.00705.x 21418511 PMC3193372

[mgg31317-bib-0025] Du, H. , Guo, L. , Yan, S. , Sosunov, A. A. , McKhann, G. M. , & Yan, S. S. (2010). Early deficits in synaptic mitochondria in an Alzheimer's disease mouse model. Proceedings of the National Academy of Sciences of the United States of America, 107, 18670–18675. 10.1073/pnas.1006586107 20937894 PMC2972922

[mgg31317-bib-0026] Eichner, J. E. , Kuller, L. H. , Orchard, T. J. , Grandits, G. A. , McCallum, L. M. , Ferrell, R. E. , & Neaton, J. D. (1993). Relation of apolipoprotein E phenotype to myocardial infarction and mortality from coronary artery disease. American Journal of Cardiology, 71, 160–165. 10.1016/0002-9149(93)90732-r 8421977

[mgg31317-bib-0027] Elias‐Sonnenschein, L. S. , Helisalmi, S. , Natunen, T. , Hall, A. , Paajanen, T. , Herukka, S. K. , … Hiltunen, M. (2013). Genetic loci associated with Alzheimer's disease and cerebrospinal fluid biomarkers in a Finnish case‐control cohort. PLoS ONE, 8, e59676 10.1371/journal.pone.0059676 23573206 PMC3616106

[mgg31317-bib-0028] Esterbauer, H. , Schneitler, C. , Oberkofler, H. , Ebenbichler, C. , Paulweber, B. , Sandhofer, F. , … Patsch, W. (2001). A common polymorphism in the promoter of UCP2 is associated with decreased risk of obesity in middle‐aged humans. Nature Genetics, 28, 178–183. 10.1038/88911 11381268

[mgg31317-bib-0029] Farre, D. , Roset, R. , Huerta, M. , Adsuara, J. E. , Rosello, L. , Alba, M. M. , & Messeguer, X. (2003). Identification of patterns in biological sequences at the ALGGEN server: PROMO and MALGEN. Nucleic Acids Research, 31, 3651–3653. 10.1093/nar/gkg605 12824386 PMC169011

[mgg31317-bib-0030] Ferencz, B. , Laukka, E. J. , Lövdén, M. , Kalpouzos, G. , Keller, L. , Graff, C. , … Bäckman, L. (2013). The influence of APOE and TOMM40 polymorphisms on hippocampal volume and episodic memory in old age. Frontiers in Human Neuroscience, 7, 198. 23734114 10.3389/fnhum.2013.00198 PMC3660657

[mgg31317-bib-0031] Gatz, M. , Reynolds, C. A. , Fratiglioni, L. , Johansson, B. , Mortimer, J. A. , Berg, S. , … Pedersen, N. L. (2006). Role of genes and environments for explaining Alzheimer disease. Archives of General Psychiatry, 63, 168–174. 10.1001/archpsyc.63.2.168 16461860

[mgg31317-bib-0032] Hansson Petersen, C. A. , Alikhani, N. , Behbahani, H. , Wiehager, B. , Pavlov, P. F. , Alafuzoff, I. , … Ankarcrona, M. (2008). The amyloid beta‐peptide is imported into mitochondria via the TOM import machinery and localized to mitochondrial cristae. Proceedings of the National Academy of Sciences of the United States of America, 105, 13145–13150. 10.1073/pnas.0806192105 18757748 PMC2527349

[mgg31317-bib-0033] Hardy, J. , & Selkoe, D. J. (2002). The amyloid hypothesis of Alzheimer's disease: Progress and problems on the road to therapeutics. Science, 297, 353–356. 10.1126/science.1072994 12130773

[mgg31317-bib-0034] He, Y. , Li, C. , Yang, Y. , Li, Y. , Wang, Y. , Yang, H. , … Chen, S. (2016). Meta‐analysis of the rs2075650 polymorphism and risk of Alzheimer disease. Aging Clinical and Experimental Research, 28, 805–811. 10.1007/s40520-015-0489-y 26572157

[mgg31317-bib-0035] Hernandez‐Zimbron, L. F. , Luna‐Munoz, J. , Mena, R. , Vazquez‐Ramirez, R. , Kubli‐Garfias, C. , Cribbs, D. H. , … Gevorkian, G. (2012). Amyloid‐beta peptide binds to cytochrome C oxidase subunit 1. PLoS ONE, 7, e42344 10.1371/journal.pone.0042344 22927926 PMC3424232

[mgg31317-bib-0036] Hinney, A. , Albayrak, O. , Antel, J. , Volckmar, A. L. , Sims, R. , Chapman, J. , … Hebebrand, J. (2014). Genetic variation at the CELF1 (CUGBP, elav‐like family member 1 gene) locus is genome‐wide associated with Alzheimer's disease and obesity. American Journal of Medical Genetics. Part B, Neuropsychiatric Genetics, 165b, 283–293. 10.1002/ajmg.b.32234 24788522

[mgg31317-bib-0037] Holtzman, D. M. , Herz, J. , & Bu, G. (2012). Apolipoprotein E and apolipoprotein E receptors: Normal biology and roles in Alzheimer disease. Cold Spring Harbor Perspectives in Medicine, 2, a006312 10.1101/cshperspect.a006312 22393530 PMC3282491

[mgg31317-bib-0038] Hong, M. G. , Alexeyenko, A. , Lambert, J. C. , Amouyel, P. , & Prince, J. A. (2010). Genome‐wide pathway analysis implicates intracellular transmembrane protein transport in Alzheimer disease. Journal of Human Genetics, 55, 707–709. 10.1038/jhg.2010.92 20668461

[mgg31317-bib-0039] Horwitz, T. , Lam, K. , Chen, Y. , Xia, Y. , & Liu, C. (2019). A decade in psychiatric GWAS research. Molecular Psychiatry, 24, 378–389. 10.1038/s41380-018-0055-z 29942042 PMC6372350

[mgg31317-bib-0040] Hu, W. , Wang, Z. , & Zheng, H. (2018). Mitochondrial accumulation of amyloid beta (Abeta) peptides requires TOMM22 as a main Abeta receptor in yeast. Journal of Biological Chemistry, 293, 12681–12689. 10.1074/jbc.ra118.002713 29925587 PMC6102147

[mgg31317-bib-0041] Huang, H. , Zhao, J. , Xu, B. , Ma, X. , Dai, Q. , Li, T. , … Chen, B. (2016). The TOMM40 gene rs2075650 polymorphism contributes to Alzheimer's disease in Caucasian, and Asian populations. Neuroscience Letters, 628, 142–146. 10.1016/j.neulet.2016.05.050 27328316

[mgg31317-bib-0042] Hyman, B. T. , Phelps, C. H. , Beach, T. G. , Bigio, E. H. , Cairns, N. J. , Carrillo, M. C. , … Montine, T. J. (2012). National Institute on Aging‐Alzheimer's Association guidelines for the neuropathologic assessment of Alzheimer's disease. Alzheimer's & Dementia, 8, 1–13. 10.1016/j.jalz.2011.10.007 PMC3266529 22265587

[mgg31317-bib-0043] Hyman, B. T. , & Trojanowski, J. Q. (1997). Consensus recommendations for the postmortem diagnosis of Alzheimer disease from the National Institute on Aging and the Reagan Institute Working Group on diagnostic criteria for the neuropathological assessment of Alzheimer disease. Journal of Neuropathology and Experimental Neurology, 56, 1095–1097. 10.1097/00005072-199710000-00002 9329452

[mgg31317-bib-0044] Iijima‐Ando, K. , Sekiya, M. , Maruko‐Otake, A. , Ohtake, Y. , Suzuki, E. , Lu, B. , & Iijima, K. M. (2012). Loss of axonal mitochondria promotes tau‐mediated neurodegeneration and Alzheimer's disease‐related tau phosphorylation via PAR‐1. PLoS Genetics, 8, e1002918 10.1371/journal.pgen.1002918 22952452 PMC3431335

[mgg31317-bib-0045] Johnson, S. C. , la Rue, A. , Hermann, B. P. , Xu, G. , Koscik, R. L. , Jonaitis, E. M. , … Sager, M. A. (2011). The effect of TOMM40 poly‐T length on gray matter volume and cognition in middle‐aged persons with APOE epsilon3/epsilon3 genotype. Alzheimer's & Dementia, 7, 456–465. 10.1016/j.jalz.2010.11.012 PMC3143375 21784354

[mgg31317-bib-0046] Jun, G. , Vardarajan, B. N. , Buros, J. , Yu, C. E. , Hawk, M. V. , Dombroski, B. A. , … Farrer, L. A. (2012). Comprehensive search for Alzheimer disease susceptibility loci in the APOE region. Archives of Neurology, 69, 1270–1279. 10.1001/archneurol.2012.2052 22869155 PMC3579659

[mgg31317-bib-0047] Khan, A. , Fornes, O. , Stigliani, A. , Gheorghe, M. , Castro‐Mondragon, J. A. , van der Lee, R. , … Mathelier, A. (2018). JASPAR 2018: Update of the open‐access database of transcription factor binding profiles and its web framework. Nucleic Acids Research, 46, D260–D266. 10.1093/nar/gkx1126 29140473 PMC5753243

[mgg31317-bib-0048] Khankhanian, P. , Gourraud, P. A. , Lizee, A. , & Goodin, D. S. (2015). Haplotype‐based approach to known MS‐associated regions increases the amount of explained risk. Journal of Medical Genetics, 52, 587–594. 10.1136/jmedgenet-2015-103071 26185143 PMC4552900

[mgg31317-bib-0049] Kondadi, A. K. , Wang, S. , Montagner, S. , Kladt, N. , Korwitz, A. , Martinelli, P. , … Rugarli, E. I. (2014). Loss of the m‐AAA protease subunit AFG(3)L(2) causes mitochondrial transport defects and tau hyperphosphorylation. EMBO Journal, 33, 1011–1026. 10.1002/embj.201387009 24681487 PMC4193934

[mgg31317-bib-0050] Kraja, A. T. , Chasman, D. I. , North, K. E. , Reiner, A. P. , Yanek, L. R. , Kilpelainen, T. O. , … Borecki, I. B. (2014). Pleiotropic genes for metabolic syndrome and inflammation. Molecular Genetics and Metabolism, 112, 317–338. 24981077 10.1016/j.ymgme.2014.04.007 PMC4122618

[mgg31317-bib-0051] Kulminski, A. M. , Loika, Y. , Culminskaya, I. , Huang, J. , Arbeev, K. G. , Bagley, O. , … Yashin, A. I. (2019). Independent associations of TOMM40 and APOE variants with body mass index. Aging Cell, 18, e12869. 30462377 10.1111/acel.12869 PMC6351823

[mgg31317-bib-0052] Li, G. , Bekris, L. M. , Leong, L. , Steinbart, E. J. , Shofer, J. B. , Crane, P. K. , … Yu, C. E. (2013). TOMM40 intron 6 poly‐T length, age at onset, and neuropathology of AD in individuals with APOE epsilon3/epsilon3. Alzheimer's & Dementia, 9, 554–561. 10.1016/j.jalz.2012.06.009 PMC3606272 23183136

[mgg31317-bib-0053] Li, H. , Wetten, S. , Li, L. , St Jean, P. L. , Upmanyu, R. , Surh, L. , … Roses, A. D. (2008). Candidate single‐nucleotide polymorphisms from a genomewide association study of Alzheimer disease. Archives of Neurology, 65, 45–53. 17998437 10.1001/archneurol.2007.3

[mgg31317-bib-0054] Lin, R. , Zhang, Y. , Yan, D. , Liao, X. , Gong, G. , Hu, J. , … Cai, W. (2016). Association of common variants in TOMM40/APOE/APOC1 region with human longevity in a Chinese population. Journal of Human Genetics, 61, 323–328. 10.1038/jhg.2015.150 26657933

[mgg31317-bib-0055] Lu, F. , Guan, H. , Gong, B. , Liu, X. , Zhu, R. , Wang, Y. , … Yang, Z. (2014). Genetic variants in PVRL2‐TOMM40‐APOE region are associated with human longevity in a Han Chinese population. PLoS ONE, 9, e99580 10.1371/journal.pone.0099580 24924924 PMC4055715

[mgg31317-bib-0056] Lyall, D. M. , Harris, S. E. , Bastin, M. E. , Munoz Maniega, S. , Murray, C. , Lutz, M. W. , … Deary, I. J. (2014). Are APOE varepsilon genotype and TOMM40 poly‐T repeat length associations with cognitive ageing mediated by brain white matter tract integrity? Transl Psychiatry, 4, e449 10.1038/tp.2014.89 25247594 PMC4203017

[mgg31317-bib-0057] Ma, X. Y. , Yu, J. T. , Wang, W. , Wang, H. F. , Liu, Q. Y. , Zhang, W. , & Tan, L. (2013). Association of TOMM40 polymorphisms with late‐onset Alzheimer's disease in a Northern Han Chinese population. NeuroMolecular Medicine, 15, 279–287. 10.1007/s12017-012-8217-7 23288655

[mgg31317-bib-0058] Manczak, M. , Anekonda, T. S. , Henson, E. , Park, B. S. , Quinn, J. , & Reddy, P. H. (2006). Mitochondria are a direct site of A beta accumulation in Alzheimer's disease neurons: Implications for free radical generation and oxidative damage in disease progression. Human Molecular Genetics, 15, 1437–1449. 10.1093/hmg/ddl066 16551656

[mgg31317-bib-0059] Marioni, R. E. , Harris, S. E. , Zhang, Q. , McRae, A. F. , Hagenaars, S. P. , Hill, W. D. , … Visscher, P. M. (2018). GWAS on family history of Alzheimer's disease. Translational Psychiatry, 8, 99. 29777097 10.1038/s41398-018-0150-6 PMC5959890

[mgg31317-bib-0060] McFarquhar, M. , Elliott, R. , McKie, S. , Thomas, E. , Downey, D. , Mekli, K. , … Juhasz, G. (2014). TOMM40 rs2075650 may represent a new candidate gene for vulnerability to major depressive disorder. Neuropsychopharmacology, 39, 1743–1753. 10.1038/npp.2014.22 24549102 PMC4023148

[mgg31317-bib-0061] Moon, S. W. , Dinov, I. D. , Kim, J. , Zamanyan, A. , Hobel, S. , Thompson, P. M. , & Toga, A. W. (2015). Structural Neuroimaging Genetics Interactions in Alzheimer's disease. Journal of Alzheimer's Disease, 48, 1051–1063. 10.3233/jad-150335 PMC4730943 26444770

[mgg31317-bib-0062] Omoumi, A. , Fok, A. , Greenwood, T. , Sadovnick, A. D. , Feldman, H. H. , & Hsiung, G. Y. (2014). Evaluation of late‐onset Alzheimer disease genetic susceptibility risks in a Canadian population. Neurobiology of Aging, 35, 936.e5–936.e12. 10.1016/j.neurobiolaging.2013.09.025 24176626

[mgg31317-bib-0063] Omoumi, A. , Fok, A. , Greenwood, T. , Sadovnick, A. D. , Feldman, H. H. , & Hsiung, G. Y. (2014). Evaluation of late‐onset Alzheimer disease genetic susceptibility risks in a Canadian population. Neurobiology of Aging, 35, 936.e5–936.e12. 10.1016/j.neurobiolaging.2013.09.025 24176626

[mgg31317-bib-0064] Potkin, S. G. , Guffanti, G. , Lakatos, A. , Turner, J. A. , Kruggel, F. , Fallon, J. H. , … Macciardi, F. ; ALZHEIMER'S DISEASE NEUROIMAGING, I . (2009). Hippocampal atrophy as a quantitative trait in a genome‐wide association study identifying novel susceptibility genes for Alzheimer's disease. PLoS ONE, 4, e6501 10.1371/journal.pone.0006501 19668339 PMC2719581

[mgg31317-bib-0065] Prendecki, M. , Florczak‐Wyspianska, J. , Kowalska, M. , Ilkowski, J. , Grzelak, T. , Bialas, K. , … Dorszewska, J. (2018). Biothiols and oxidative stress markers and polymorphisms of TOMM40 and APOC1 genes in Alzheimer's disease patients. Oncotarget, 9, 35207–35225. 10.18632/oncotarget.26184 30443289 PMC6219666

[mgg31317-bib-0066] Querfurth, H. W. , & Laferla, F. M. (2010). Alzheimer's disease. New England Journal of Medicine, 362, 329–344. 20107219 10.1056/NEJMra0909142

[mgg31317-bib-0067] Ray, S. K. , Nishitani, J. , Petry, M. W. , Fessing, M. Y. , & Leiter, A. B. (2003). Novel transcriptional potentiation of BETA2/NeuroD on the secretin gene promoter by the DNA‐binding protein Finb/RREB‐1. Molecular and Cellular Biology, 23, 259–271. 10.1128/mcb.23.1.259-271.2003 12482979 PMC140679

[mgg31317-bib-0068] Rojo, A. I. , Pajares, M. , Rada, P. , Nunez, A. , Nevado‐Holgado, A. J. , Killik, R. , … Cuadrado, A. (2017). NRF2 deficiency replicates transcriptomic changes in Alzheimer's patients and worsens APP and TAU pathology. Redox Biology, 13, 444–451. 10.1016/j.redox.2017.07.006 28704727 PMC5508523

[mgg31317-bib-0069] Roses, A. D. , Lutz, M. W. , Amrine‐Madsen, H. , Saunders, A. M. , Crenshaw, D. G. , Sundseth, S. S. , … Reiman, E. M. (2010). A TOMM40 variable‐length polymorphism predicts the age of late‐onset Alzheimer's disease. The Pharmacogenomics Journal, 10, 375–384. 10.1038/tpj.2009.69 20029386 PMC2946560

[mgg31317-bib-0070] Scheltens, P. , Blennow, K. , Breteler, M. M. , de Strooper, B. , Frisoni, G. B. , Salloway, S. , & van der Flier, W. M. (2016). Alzheimer's disease. Lancet, 388, 505–517. 26921134 10.1016/S0140-6736(15)01124-1

[mgg31317-bib-0071] Schott, J. M. (2012). Using CSF biomarkers to replicate genetic associations in Alzheimer's disease. Neurobiology of Aging, 33, 1486.e9–1486.e15. 10.1016/j.neurobiolaging.2011.02.008 PMC3150628 21459483

[mgg31317-bib-0072] Schupf, N. , Barral, S. , Perls, T. , Newman, A. , Christensen, K. , Thyagarajan, B. , … Mayeux, R. (2013). Apolipoprotein E and familial longevity. Neurobiology of Aging, 34, 1287–1291. 10.1016/j.neurobiolaging.2012.08.019 23040522 PMC3545094

[mgg31317-bib-0073] Selkoe, D. J. (2011). Alzheimer's disease. Cold Spring Harbor Perspectives in Biology, 3(7), a004457 10.1101/cshperspect.a004457 21576255 PMC3119915

[mgg31317-bib-0074] Seripa, D. , Bizzarro, A. , Pilotto, A. , Palmieri, O. , Panza, F. , D'Onofrio, G. , … Masullo, C. (2012). TOMM40, APOE, and APOC1 in primary progressive aphasia and frontotemporal dementia. Journal of Alzheimer's Disease, 31, 731–740. 10.3233/jad-2012-120403 22710912

[mgg31317-bib-0075] Shadyab, A. H. , Kooperberg, C. , Reiner, A. P. , Jain, S. , Manson, J. E. , Hohensee, C. , … Lacroix, A. Z. (2017). Replication of genome‐wide association study findings of longevity in White, African American, and Hispanic Women: The women's health initiative. Journals of Gerontology. Series A, Biological Sciences and Medical Sciences, 72, 1401–1406. 10.1093/gerona/glw198 PMC5861976 27707806

[mgg31317-bib-0076] Shao, Y. , Shaw, M. , Todd, K. , Khrestian, M. , D'Aleo, G. , Barnard, P. J. , … Bekris, L. M. (2018). DNA methylation of TOMM40‐APOE‐APOC2 in Alzheimer’s disease. Journal of Human Genetics, 63(4), 459–471. 10.1038/s10038-017-0393-8 29371683 PMC6466631

[mgg31317-bib-0077] Sheng, Z. H. (2014). Mitochondrial trafficking and anchoring in neurons: New insight and implications. Journal of Cell Biology, 204, 1087–1098. 10.1083/jcb.201312123 24687278 PMC3971748

[mgg31317-bib-0078] Shi, H. , Belbin, O. , Medway, C. , Brown, K. , Kalsheker, N. , Carrasquillo, M. , … Morgan, K. (2012). Genetic variants influencing human aging from late‐onset Alzheimer's disease (LOAD) genome‐wide association studies (GWAS). Neurobiology of Aging, 33, 1849.e5–1849.e18. 10.1016/j.neurobiolaging.2012.02.014 PMC4120742 22445811

[mgg31317-bib-0079] Shim, H. , Chun, H. , Engelman, C. D. , & Payseur, B. A. (2009). Genome‐wide association studies using single‐nucleotide polymorphisms versus haplotypes: An empirical comparison with data from the North American Rheumatoid Arthritis Consortium. BMC Proceedings, 3(Suppl 7), S35 10.1186/1753-6561-3-s7-s35 20018026 PMC2795933

[mgg31317-bib-0080] Shiota, T. , Imai, K. , Qiu, J. , Hewitt, V. L. , Tan, K. , Shen, H. H. , … Endo, T. (2015). Molecular architecture of the active mitochondrial protein gate. Science, 349, 1544–1548. 10.1126/science.aac6428 26404837

[mgg31317-bib-0081] Souza, M. B. , Araújo, G. S. , Costa, I. G. , & Oliveira, J. R. (2016). Combined genome‐wide CSF Aβ‐42's associations and simple network properties highlight new risk factors for Alzheimer's disease. Journal of Molecular Neuroscience, 58, 120–128. 10.1007/s12031-015-0667-6 26576771

[mgg31317-bib-0082] Stancu, I. C. , Vasconcelos, B. , Terwel, D. , & Dewachter, I. (2014). Models of beta‐amyloid induced Tau‐pathology: The long and "folded" road to understand the mechanism. Molecular Neurodegeneration, 9, 51 10.1186/1750-1326-9-51 25407337 PMC4255655

[mgg31317-bib-0083] Strittmatter, W. J. , Saunders, A. M. , Schmechel, D. , Pericak‐Vance, M. , Enghild, J. , Salvesen, G. S. , & Roses, A. D. (1993). Apolipoprotein E: High‐avidity binding to beta‐amyloid and increased frequency of type 4 allele in late‐onset familial Alzheimer disease. Proceedings of the National Academy of Sciences of the United States of America, 90, 1977–1981. 10.1073/pnas.90.5.1977 8446617 PMC46003

[mgg31317-bib-0084] Wijsman, E. M. , Pankratz, N. D. , Choi, Y. , Rothstein, J. H. , Faber, K. M. , Cheng, R. , … Mayeux, R. (2011). Genome‐wide association of familial late‐onset Alzheimer's disease replicates BIN1 and CLU and nominates CUGBP2 in interaction with APOE. PLoS Genetics, 7, e1001308 10.1371/journal.pgen.1001308 21379329 PMC3040659

[mgg31317-bib-0085] Xiao, Q. , Liu, Z. J. , Tao, S. , Sun, Y. M. , Jiang, D. , Li, H. L. , … Wu, Z. Y. (2015). Risk prediction for sporadic Alzheimer's disease using genetic risk score in the Han Chinese population. Aging Clinical and Experimental Research, 6, 36955–36964. 10.18632/oncotarget.6271 PMC4741908 26543236

[mgg31317-bib-0086] Xu, Z. , Shen, X. , & Pan, W. (2014). Longitudinal analysis is more powerful than cross‐sectional analysis in detecting genetic association with neuroimaging phenotypes. PLoS ONE, 9, e102312 10.1371/journal.pone.0102312 25098835 PMC4123854

[mgg31317-bib-0087] Yamase, Y. , Horibe, H. , Ueyama, C. , Fujimaki, T. , Oguri, M. , Kato, K. , … Yamada, Y. (2015). Association of TOMM40 and SLC22A4 polymorphisms with ischemic stroke. Biomed Rep, 3, 491–498. 10.3892/br.2015.457 26171154 PMC4487021

[mgg31317-bib-0088] Yu, L. , Lutz, M. W. , Wilson, R. S. , Burns, D. K. , Roses, A. D. , Saunders, A. M. , … Bennett, D. A. (2017). TOMM40'523 variant and cognitive decline in older persons with APOE epsilon3/3 genotype. Neurology, 88, 661–668. 28108637 10.1212/WNL.0000000000003614 PMC5317377

