# Supplementary material for: A TOMM40/APOE allele encoding APOE‐E3 predicts high likelihood of late‐onset Alzheimer’s disease in autopsy cases
Source: Mol Genet Genomic Med. 2020 May 30;8(8):e1317. doi: 10.1002/mgg3.1317 (PMC7434743; doi:10.1002/mgg3.1317)
Supplement: Supplementary file 1 — Table S1‐S8 [file MGG3-8-e1317-s001.docx]

# Supplementary Material, Tables S1 – S8

**Table S1**. Matrix of pairwise standardized linkage disequilibria (D’) and R^2^-values

|  | rs157580 | rs2075650 | rs8106922 | rs429358 | rs7412 |
| --- | --- | --- | --- | --- | --- |
| rs157580 |  | -0.90 (0.000) | -0.93 (0.000) | -1.00 (0.000) | 0.14 (0.091) |
| rs2075650 | 0.0452 (0.0002) |  | -0.85 (0.000) | 0.60 (0.000) | -1.00 (0.001) |
| rs8106922 | 0.3149(0.0000) | .1361 (0.0000) |  | -1.00 (0.000) | -0.78(0.000) |
| rs429358 | .0591 (0.0000) | .3537 (0.0000) | .1340 (0.0000) |  | -1.0(0.001) |
| rs7412 | .0146 (.0385) | .0361 (.0011) | .0473 (.0002) | .0253(.0063) |  |

Pairwise LD in terms of D’ (upper right half) and R^2^ (lower left half) with *p*-values in parentheses.

# Table S2. *APOE* genotypes and associated risk of AD

|  |  |  |  |  |  |  | |
| --- | --- | --- | --- | --- | --- | --- | --- |
|  | Controls | | AD Cases | |  | Odds ratio (95% CI) | |
|  |  |  |  |  |  |  |  |
| Genotype | N | Frequency | N | Frequency | P^a^ | Univariate analysis^b^ | Multivariate analysis^c^ |
|  |  |  |  |  |  |  |  |
| 33 | 99 | 0.6600 | 73 | 0.5069 |  | 1.00 | 1.00 |
| 22  23 | 2 | 0.0133 | 1 | 0.0069 |  | 0.68 (0.06-7.62) | 0.89 (0.04-18.70) |
| 23 | 27 | 0.1800 | 18 | 0.1250 |  | 0.90 (0.46-1.76) | 0.72 (0.33-1.56) |
| 24 | 0 | 0 | 6 | 0.0417 |  | * | * |
| 34 | 21 | 0.1400 | 38 | 0.2639 |  | 2.45 (1.33-4.53) | 4.54 (2.05-10.06) |
| 44 | 1 | 0.0067 | 8 | 0.0556 | 0.00046 | 10.08 (1.33-88.66) | 27.84 (2.90-267.39) |

^a^ **χ^2^** analysis; ^b^ logistic regression; ^c^ logistic regression adjusted for sex and age; * genotype absent in controls.

# Table S3. *TOMM40* rs2075650 and associated risk of AD in *APOE*-ε3/3 genotypes

|  |  |  |  |  |  |  |  |
| --- | --- | --- | --- | --- | --- | --- | --- |
|  | Controls | | AD Cases | |  | Odds Ratio (95% CI) | |
|  |  |  |  |  |  |  |  |
| Genotype | N = 99 | Frequency | N = 73 | Frequency | P^a^ | Univariate analysis^b^ | Multivariate analysis^c^ |
|  |  |  |  |  |  |  |  |
| AA | 91 | 0.9192 | 57 | 0.7808 |  | 1.00 | 1.00 |
| AG | 7 | 0.0707 | 14 | 0.1918 |  | 3.19 (1.21-8.39) | 2.31 (0.76-7.04) |
| GG | 1 | 0.0101 | 2 | 0.0274 | 0.0351 | 3.19 (0.28-36.02) | 1.16 (0.10-13.61) |

# ^a^ χ^2^ analysis; ^b^ logistic regression; ^c^ logistic regression adjusted for sex and age.

# Table S4. Cases with intermediate- and high-likelihood of AD

|  |  |  |  |
| --- | --- | --- | --- |
| Variable | Intermediate likelihood AD | High likelihood AD | *P* |
|  |  |  |  |
| Sex, male/females | 31/42 | 35/36 | n.s. |
| Age of death, yrs | 84.5 (6.7) | 86.6 (5.7) | 0.0467 |
| BRAAK, 0 - I/II – III/IV – V/VI | 0/14/59/0 | 0/0/0/71 | n.d. |
| CERAD, 0 – A.- B –C | 0/15/35/23 | 0/2/14/55 | 0.00011 |

# Table S5. Polymorphisms and associated risk of intermediate-likelihood AD

|  |  |  | |  | |  |  | |
| --- | --- | --- | --- | --- | --- | --- | --- | --- |
|  |  | Intermediate likelihood AD cases | | Controls | |  | Odds ratio (95% CI) | |
|  |  |  |  |  |  |  |  |  |
| Gene/SNP | Genotype | N | Frequency | N | Frequency | P^a^ | Univariate analysis^b^ | Multivariate analysis^c^ |
|  |  |  |  |  |  |  |  |  |
| *TOMM40*/rs157580 | AA | 30 | 0.4110 | 63 | 0.4200 |  | 1.00 | 1.00 |
|  | AG | 35 | 0.4795 | 74 | 0.4933 |  | 0.99 (0.55-1.80) | 1.29 (0.67-2.51) |
|  | GG | 8 | 0.1096 | 13 | 0.0867 | 0.8594 | 1.29 (0.48-3.45) | 1.02 (0.34-3.08) |
|  |  |  |  |  |  |  |  |  |
| *TOMM40/*rs2075650 | AA | 52 | 0.7123 | 123 | 0.8200 |  | 1.00 | 1.00 |
|  | AG | 19 | 0.2603 | 26 | 0.1733 |  | 1.72 (0.88-3.39) | 2.25 (1.05-4.84) |
|  | GG | 2 | 0.0274 | 1 | 0.0067 | 0.1266 | 4.73 (0.42-53.33) | 8.21 (0.56-117.36) |
|  |  |  |  |  |  |  |  |  |
| *TOMM40/*rs8106922 | AA | 27 | 0.3699 | 44 | 0.2933 |  | 1.00 | 1.00 |
|  | AG | 34 | 0.4658 | 69 | 0.4600 |  | 0.80 (0.43-1.51) | 0.72 (0.35-1.48) |
|  | GG | 12 | 0.1644 | 37 | 0.2467 | 0.2982 | 0.53 (0.24-1.19) | 0.34 (0.15-0.88) |
|  |  |  |  |  |  |  |  |  |
| *APOE*/rs429358 | TT | 47 | 0.6438 | 128 | 0.8533 |  | 1.00 | 1.00 |
|  | TC | 24 | 0.3288  512 | 21 | 0.1400 |  | 3.11 (1.59-6.11) | 6.94 (2.90-16.64) |
|  | CC | 2 | 0.0274 | 1 | 0.0067 | 0.0015 | 5.45 (0.48-61.48) | 22.39 (1.67-299.28) |
|  |  |  |  |  |  |  |  |  |
| *APOE*/rs7412 | CC | 57 | 0.7808 | 121 | 0.8067 |  | 1.00 | 1.00 |
|  | CT | 15 | 0.2055 | 27 | 0.1800 |  | 1.18 (0.58-2.39) | 0.85 (0.39-1.85) |
|  | TT | 1 | 0.0137 | 2 | 0.0133 | 0.8999 | 1.06 (0.09-11.94) | 1.10 (0.06-18.85) |

^a^ **χ^2^** analysis; ^b^ logistic regression; ^c^ logistic regression adjusted for sex and age.

**Table S6** Polymorphisms and associated risk of high likelihood for AD

|  |  |  | |  | |  |  | |
| --- | --- | --- | --- | --- | --- | --- | --- | --- |
|  |  | High Likelihood AD Cases | | Controls | |  | Odds ratio (95% CI) | |
|  |  |  |  |  |  |  |  |  |
| GENE/SNP | Genotype | N | Frequency | N | Frequency | P^a^ | Univariate analysis^b^ | Multivariate analysis^c^ |
|  |  |  |  |  |  |  |  |  |
| *TOMM40*/rs157580 | AA | 40 | 0.5634 | 63 | 0.4200 |  | 1.00 | 1.00 |
|  | AG | 29 | 0.4085 | 74 | 0.4933 |  | 0.59 (0.33-1.05) | 0.72 (0.37-1.42) |
|  | GG | 2 | 0.0282 | 13 | 0.0867 | 0.0706 | 0.23 (0.05-1.08) | 0.22 (0.04-1.13) |
|  |  |  |  |  |  |  |  |  |
| *TOMM40*/rs2075650 | AA | 39 | 0.5493 | 123 | 0.8200 |  | 1.00 | 1.00 |
|  | AG | 27 | 0.3803 | 26 | 0.1733 |  | 3.52 (1.85-6.67) | 3.79 (1.78-8.06) |
|  | GG | 5 | 0.0704 | 1 | 0.0067 | 0.00003 | 15.77 (1.79-139.09) 111148.521.39) | 12.55 (1.18-133.74) |
|  |  |  |  |  |  |  |  |  |
| *TOMM40*/rs8106922 | AA | 28 | 0.3944 | 44 | 0.2933 |  | 1.00 | 1.00 |
|  | AG | 32 | 0.4507 | 69 | 0.4600 |  | 0.73 (0.39-1.36) | 0.82 (0.39-1.71) |
|  | GG | 11 | 0.1549 | 37 | 0.2467 | 0.1840 | 0.44 (0.20-1.02) | 0.28 (0.11-0.71) |
|  |  |  |  |  |  |  |  |  |
| *APOE*/rs429358 | TT | 45 | 0.6338 | 128 | 0.8533 |  | 1.00 | 1.00 |
|  | TC | 20 | 0.2817  512 | 21 | 0.1400 |  | 2.98 (1.50-5.93) | 6.26 (2.48-15.81) |
|  | CC | 6 | 0.0845 | 1 | 0.0067 | 0.0002 | 17.07 (2.00-145.65) | 31.77 (3.19-316.21) |
|  |  |  |  |  |  |  |  |  |
| *APOE*/rs7412 | CC | 62 | 0.8732 | 121 | 0.8067 |  | 1.00 | 1.00 |
|  | CT | 9 | 0.1268 | 27 | 0.1800 |  | 0.63 (0.28-1.42) | 0.44 (0.18-1.08) |
|  | TT | 0 | 0.0000 | 2 | 0.0133 | 0.3600 | - | - |

# Table S7. *TOMM40* rs2075650 and associated risk of intermediate-likelihood AD in APOE-ε3/3 genotypes

|  |  |  | |  | |  |  | |
| --- | --- | --- | --- | --- | --- | --- | --- | --- |
|  |  | Cases | | Controls | |  | Odds ratio (95% CI) | |
|  |  |  |  |  |  |  |  |  |
| Genotype |  |  | Frequency | N | Frequency | P^a^ | Univariate analysis^b^ | Multivariate analysis^c^ |
|  |  |  |  |  |  |  |  |  |
|  |  |  |  |  |  |  |  |  |
| rs2075650 | AA | 31 | 0.8857 | 91 | 0.9192 |  | 1.00 | 1.00 |
|  | AG | 4 | 0.1143 | 7 | 0.0707 |  | 1.68 (0.46-6.12) | 1.41 (0.37-5.34) |
|  | GG | 0 | 0.0000 | 1 | 0.0101 | 0.6155 | - | - |

# ^a^ χ^2^ analysis; ^b^ logistic regression; ^c^ logistic regression adjusted for sex and age.

# Table S8. *TOMM40* rs2075650 and associated risk of high-likelihood AD in *APOE*-ε3/3 genotypes

|  |  |  | |  | |  |  | |
| --- | --- | --- | --- | --- | --- | --- | --- | --- |
|  |  | Cases | | Controls | |  | Odds ratio (95% CI) | |
|  |  |  |  |  |  |  |  |  |
| Genotype |  |  | Frequency | N | Frequency | p^a^ | Univariate analysis^b^ | Multivariate analysis^c^ |
|  |  |  |  |  |  |  |  |  |
|  |  |  |  |  |  |  |  |  |
| rs2075650 | AA | 26 | 0.6842 | 91 | 0.9192 |  | 1.00 | 1.00 |
|  | AG | 10 | 0.2632 | 7 | 0.0707 |  | 5.00 (1.73-14.43) | 3.65 (1.17-11.80) |
|  | GG | 2 | 0.0526 | 1 | 0.0101 | 0.0022 | 7.00 (0.61-80.27) | 3.07 (0.26-35.89) |

# ^a^ χ^2^ analysis; ^b^ logistic regression; ^c^ logistic regression adjusted for sex and age.
